# Generation of *H11-albumin-rtTA* Transgenic Mice: A Tool for Inducible Gene Expression in the Liver

**DOI:** 10.1534/g3.118.200963

**Published:** 2018-12-27

**Authors:** Yu-Shan Li, Ran-Ran Meng, Xiu Chen, Cui-Ling Shang, Hong-Bin Li, Tao-Jun Zhang, Hua-Yang Long, Hui-Qi Li, Yi-Jing Wang, Feng-Chao Wang

**Affiliations:** * The School of Public Health, Xinxiang Medical University, Xinxiang, Henan, China 453003; † Transgenic Animal Center, National Institute of Biological Sciences, Beijing, China 102206; ‡ Department of Pharmacy, Heze University, Heze, Shandong, China, 274015; § Department of Reproductive Medicine, The Third Affiliated Hospital of Xinxiang Medical University, Xinxiang, Henan, China 453003

**Keywords:** *Hipp11*, *albumin* promoter, doxycycline, Tet-on system

## Abstract

The modification of the mouse genome by site-specific gene insertion of transgenes and other genetic elements allows the study of gene function in different developmental stages and in the pathogenesis of diseases. Here, we generated a “genomic safe harbor” *Hipp11* (*H11*) locus-specific knock-in transgenic mouse line in which the *albumin* promoter is used to drive the expression of the reverse tetracycline transactivator (rtTA) in the liver. The newly generated *H11-albumin-rtTA* transgenic mice were bred with *tetracycline-operator-Histone-2B-green fluorescent protein (TetO-H2BGFP*) mice to assess inducibility and tissue-specificity. Expression of the H2BGFP fusion protein was observed exclusively upon doxycycline (Dox) induction in the liver of *H11-albumin-rtTA/TetO-H2BGFP* double transgenic mice. To further analyze the ability of the Dox-inducible *H11-albumin-rtTA mice* to implement conditional DNA recombination, *H11-albumin-rtTA* transgenic mice were crossed with *TetO-Cre* and *Ai14* mice to generate *H11-albumin-rtTA/TetO-Cre/Ai14* triple transgenic mice. We successfully confirmed that the Cre-mediated recombination efficiency was as strong in Dox-induced *H11-albumin-rtTA /TetO-Cre/Ai14* mice as in the control *albumin-Cre/A14* mice. Finally, to characterize the expression-inducing effects of Dox in *H11-albumin-rtTA/TetO-H2BGFP* mice in detail, we examined GFP expression in embryos at different developmental stages and found that newly conceived *H11-albumin-rtTA/TetO-H2BGFP* embryos of Dox-treated pregnant female mice were expressing reporter GFP by E16.5. Our study demonstrates that these new *H11-albumin-rtTA* transgenic mice are a powerful and efficient tool for the temporally and spatially conditional manipulation of gene expression in the liver, and illustrates how genetic crosses with these new mice enable the generation of complex multi-locus transgenic animals for mechanistic studies.

Mouse genome modification via the stable insertion of functional transgenes and other genetic elements is currently of great significance to biological and medical research. There are two broad strategies that can be applied to generate transgenic mice: random or site-specific integration. Notably, randomly inserted genes are subject to position effects, and can result in unstable phenotypes, gene silencing, and/or unexpected gene expression (Sadelain *et al.* 2011). With site-specific gene integration, alternatively, particular locations in the host genome are selected based on particular research goals. As site-specific integration of DNA requires the identification of a “genomic safe harbor” locus that will allow for gene expression but that will not perturb surrounding endogenous gene function (Ruan *et al.* 2015). At present, the *ROSA26* locus is widely used as a transgene insertion site, owing to its ubiquitous gene expression and few reported insertion site side effects (Zambrowicz *et al.* 1997; Ma *et al.* 2017; Movahedi *et al.* 2018).

Consider that functional studies based on genetically modified mice often require the generation of complex genotypes (double-hybridization, triple-, etc.). When researchers need to make crosses with mice that already harbor engineered cassettes at their *Rosa26* locus, the ability to introduce any additional modifications would depend on the use of an alternative genomic safe harbor locus. One potential alternative safe harbor, the *H11* locus, was first described by Hippenmeyer *et al.* (2010), and has been used in human stem cells (Zhu *et al.* 2014) and in transgenic mice (Tasic *et al.* 2011). In mice, the *H11* locus occurs in an intergenic region near the chromosome 11 centromere that is positioned between the *Eif4enif1* and *Drg1* genes, and *in vivo* experiments have established that the integration of targeting cassettes from the *H11* locus does not affect mouse viability or fertility and that biallelic expression of targeting cassettes was possible (Hippenmeyer *et al.* 2010; Tasic *et al.* 2011).

Unlike the *Rosa26* locus, the *H11* locus does not have an endogenous promoter (Hippenmeyer *et al.* 2010; Ruan *et al.* 2015). We reasoned that this would make the *H11* locus particularly suitable for the conditional expression of transgenes engineered to be driven by a variety of tissue-specific promoters or otherwise inducible expression systems. The tetracycline on (Tet-on) system, which is also known as the rtTA-dependent system, has been widely-used in genetic studies to enable the conditional regulation of gene expression in a wide variety of cells, tissue cultures, and transgenic animals (Zhou *et al.* 2006; Das *et al.* 2016; Bijlani *et al.* 2018). It is composed of two elements: the ligand-dependent transactivator tetracycline rtTA as the effector and a TetO-cytomegalovirus (TetO-CMV) minimal promoter cassette regulating the expression of the transgene as the responder. In the presence of tetracycline, or one of its analogs like doxycycline (Dox), rtTA binds to TetO-sequence and activates the transcription of target genes (Burger *et al.* 2011; Cox *et al.* 2014; Thiem *et al.* 2016).

Researchers have combined the Tet-on inducible expression system with tissue-specific promoters (*e.g.*, *major urinary protein* promoter, *albumin* promoter for liver-specific expression (Gandhi *et al.* 2015)) to achieve both temporal and spatial control of the expression of genes-of-interest for functional studies in various stages of embryonic development and adulthood (Chen *et al.* 2011; Gandhi *et al.* 2015). For example, Zhou *et al.* (2012) crossed *albumin-rtTA* mice with *TetO-urokinase plasminogen activator* (*uPA*) transgenic mice to produce double transgenic *albumin-rtTA/TetO-uPA* mice in which both Dox-inducible and liver-specific over-expression of a *uPA* transgene was achieved (Zhou *et al.* 2012).

Here, seeking to expand the toolbox available for targeted knock-in experimental strategies by generating mice with an additional and highly flexible genomic safe harbor locus, we generated a new site-specific knock-in transgenic mouse model in which an *albumin-rtTA* cassette was inserted into the *H11* locus. Note that our selection of the albumin promoter also offers a new experimental system both basic and medical researchers of hepatocytes. To initially assess the inducibility and tissue-specificity of transgene expression, *H11-albumin-rtTA* transgenic mice were bred with *TetO-H2BGFP* mice to generate *H11-albumin-rtTA/TetO-H2BGFP* double transgenic mice. We confirmed that the administration of Dox induced the liver-specific expression of an *H2BGFP* reporter transgene, and cessation of Dox administration halted transgene expression in these double transgenic mice. Subsequently, *H11-albumin-rtTA* transgenic mice were crossed with *TetO-Cre* and *Ai14* mice to generate *H11-albumin-rtTA/TetO-Cre/Ai14* triple transgenic mice and we successfully confirmed very strong Dox-inducible conditional Cre-mediated recombination efficiency in these *H11-albumin-rtTA/TetO-Cre/Ai14* mice. Finally, a study of embryos conceived by Dox-treated *H11-albumin-rtTA/TetO-H2BGFP* mothers revealed that embryos were expressing the reporter by E16.5. Collectively, this study demonstrates that our *H11-albumin-rtTA* transgenic mice are a powerful and efficient tool for conditional gene expression in the liver and illustrates how crosses with these mice can generate complex genotypes to facilitate mechanistic studies.

## Materials and Methods

### H11-albumin-rtTA plasmid construction

**Figure 1:**
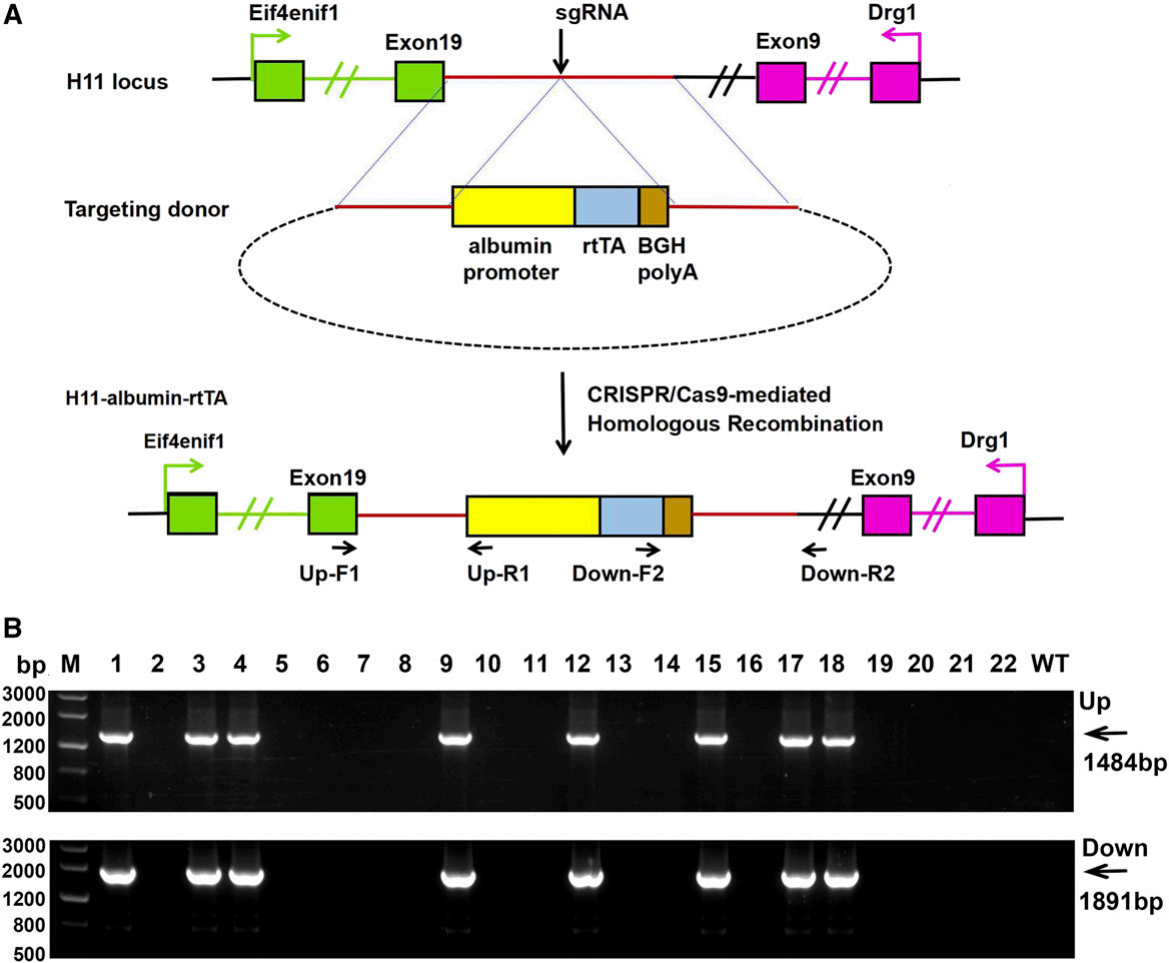
Generation of *H11-albumin-rtTA* knock-in mice using the CRISPR/Cas9 system. (A) Schematic overview of CRISPR/Cas9-mediated knock-in of the *albumin-rtTA* cassette at the *H11* locus. Top panel shows the organization of the *H11* genomic locus. Green boxes indicate exons 1 and 19 of the flanking gene *Eif4enif1*, and pink boxes indicate exons 1 and 9 of *Drg1*. The middle panel shows the design of the *H11-albumin-rtTA* targeting donor. The bottom panel shows the design for how the targeting donor is recombined into the *H11* genomic locus via CRISPR/Cas9-mediated homologous recombination in mice. (B) PCR amplification of the sgRNA:Cas9-mediated *albumin-rtTA* cassette knock-in at the endogenous *H11* locus. Upper panel: PCR amplification of the genomic junction with the left homologous arm and part of the albumin cassette. Lower panel: PCR amplification of the genomic junction with the right homologous arm and part of the rtTA cassette. Lanes 1, 3, 4, 9, 12, 15, 17, and 18 were transgene^+^ mice; lanes 2, 5, 6, 7, 8, 10, 11, 13, 14, 16, 19, 20, 21, and 22 were transgene^-^ mice. M, Marker; Up, upstream;Down, downstream.

For the liver-specific expression of rtTA at the *H11* locus, the transgenic construct *H11-albumin-rtTA* was generated. The *H11* locus homologous arms were amplified by PCR using genomic DNA extracted from a C57BL/6 mouse as the template. The *albumin* promoter and enhancer fragment was amplified by PCR using pALB-GFP as the template. A coding-sequence-improved version of the *rtTA-advanced* element was amplified by PCR using plasmid pPB-CAG-rtTA-IN as the template. The *Bovine Growth Hormone Polyadenylation Signal* (*BGHpolyA*) element was amplified by PCR using plasmid vpCRII-TOPO CMV-cGFP-BGH poly(A) as the template. All the PCR donor template sequences were cloned into the pBS plasmid using Gibson assembly reactions (E2611S/L; New England Biolabs), and incorporated into the *H11-albumin-rtTA* donor plasmid. We generated *H11-albumin-rtTA* mice via Cas9/sgRNA mediated gene targeting in zygotes (see Figure 1A, below). The primers for the donor template sequences and the sgRNA sequences used in this study are listed in the Supplemental Materials (Table S1).

### Cas9/sgRNA production and injection

The sgRNAs were prepared using a MEGAshortscript T7 Transcription kit (AM1354; Ambion) according to the manufacturer’s instructions. TheT7-Cas9 DNA was amplified by PCR using plasmid hCas9 as the template and *in vitro* transcribed using a T7 Ultra Kit (AM1345; Ambion). *Cas9* mRNA was purified using an RNeasy Mini Kit (74104; Qiagen). For microinjection, the *Cas9* mRNA and sgRNAs were diluted into injection buffer (0.25 mM EDTA/10 mM TrisHCl, pH 7.4) and incubated for 10 min at 37° before injection. The final concentration of *Cas9* was 80 ng/µl, and that of sgRNA was 30 ng/µl. The *H11-albumin-rtTA* donor plasmid concentration was 10 ng/μl for the injection.

The microinjection process was performed as described previously (Yang *et al.* 2014). Briefly, zygotes were obtained from female donor mice mated with C57BL/6 males after treatment with pregnant mare serum gonadotropin (PMSG; Sigma-Aldrich) and human chorionic gonadotropin (hCG; Sigma-Aldrich). Microinjection was performed under an inverted microscope (OLYMPUS IX71) equipped with a microinjector system (Eppendorf FemtoJet 4i). Microinjections were into the larger male pronucleus of fertilized oocytes. After microinjection, the injected zygotes were transferred to pseudopregnant C57BL/6 mice (20-30 zygotes per pseudopregnant mice). The primers used for genotyping the *H11-albumin-rtTA* mice are listed in Supplemental Materials, Table S2.

### Mouse lines and Dox treatment

*Ai14* (*Rosa26-CAG-loxp-stop-loxp-tdTomato*) (Guenthner *et al.* 2013), *TetO-Cre* (Uemura *et al.* 2014), *TetO-H2BGFP* (Chakkalakal *et al.* 2012) and *Albumin-Cre* (Cui *et al.* 2016) mice were obtained from The Jackson Laboratory. All mice were housed in an SPF environment. This study was carried out in accordance with the Animal Care and Use Committee of the National Institute of Biological Sciences, Beijing, which follow the governmental regulations of China. Adult mice were 6-to 8-weeks-old for this study. The age of mouse embryos was determined by the appearance of the vaginal plug, which was taken to be E0.5. The birth day of new pups was denoted as P1 for these experiments.

To examine how Dox treatment affected transgene expression, we administrated the tetracycline derivative Dox (D9891; Sigma) in the adult mouse drinking water at a concentration of 1 mg/ml for 48 h and then ceased treatment. Dox was dissolved in 5% sucrose (pH 6.0) to mask the bitter taste. It was kept in aluminum foil-wrapped bottles to prevent light-induced degradation.

### Western blotting

Livers were lysed with RIPA buffer (9806; Cell Signaling) containing 1 mM phenylmethylsulfonyl fluoride (8553S; Cell Signaling). The protein concentration of each sample was determined using a bicinchoninic acid (BCA) assay reagent (Vigorous Biotechnology) according to the manufacturer’s recommendations. An equal amount of each protein sample was electrophoresed on a 10% acrylamide gel and the bands were then transferred onto polyvinylidene difluoride (PVDF) membranes (Bio-Rad). The membrane was blocked with 5% non-fat dry milk for 3 h and incubated with GFP antibody (#2555; Cell Signaling) and internal control β-actin antibody (ab8227; Abcam) overnight at 4°. The PVDF membrane was then washed three times for 30 min in 0.1% Tween-20 in Tris-buffered saline (TBST) and incubated for 1 h with horseradish peroxidase-conjugated goat anti-rabbit IgG (Zhongshan). After washing for 30 min with three changes of TBST, the membrane was treated with the Pierce ECL 2 western blotting Substrate (Thermo Scientific).

### Hematoxylin and eosin (HE) staining

Hematoxylin and eosin staining was performed as previously described (Li *et al.* 2014). Briefly, sections were dewaxed, rehydrated, stained with hematoxylin, incubated in bluing solution, counterstained with eosin, dehydrated, and equilibrated with xylene. Glass coverslips were mounted with Permount Mounting Media (SP15-100; ThermoFisher Scientific). Sections were photographed using a bright-field microscope system (Leica Microsystems).

### Immunofluorescence

Liver were fixed in 4% paraformaldehyde (PFA) overnight, and then dehydrated in 30% sucrose/PBS. Tissue was embedded in Cryo-gel OCT compound (62806-01; Tissue-Tek) and frozen on dry ice. Fixed livers were sectioned at 10 μm using cryostat. Cryosections were washed 5 min×3 in PBS, counterstained with DAPI (10236276001; Roche Applied Science) for 10 min, and mounted on plus-coated slides that were cover-slipped using Vectashield (H-1000; Vector Laboratories). Finally, sections were photographed under a fluorescence microscope (Leica Microsystems).

For the labeling of albumin, cryosections were pretreated as above. Sections were blocked using 10% donkey serum in PBS, and incubated with anti-albumin antibody (Ab207327; Abcam) overnight at 4°. Subsequently, sections were washed and incubated with DAR-555 (A-31572; ThermoFisher Scientific) at 37° for 1 h.

### PCR and quantitative real-time PCR (qRT-PCR)

Genomic DNA was isolated from tail biopsies following the HotSHOT method (Truett *et al.* 2000) and genotyping was performed using standard PCR methods with sequence-specific primers (listed in Supplemental Materials, Table S3). Total RNA was extracted from the liver and various tissues using Trizol reagent (Vigorous Biotechnology) according to the manufacturer’s protocols. RNA was converted to cDNA using M-MLV reverse transcriptase (M170A; Promega) according to the manufacturer’s protocols. qRT-PCR was performed with SYBR Green master mix (DRR420A; Takara) using an ABI PRISM 7500 Sequence Detection System (Applied Biosystems). Relative RNA quantifications were normalized to the endogenous control (*Gapdh*). Data were analyzed using the 2^–△△Ct^ method. All of the experiments were repeated independently at least three times. The primers used are listed in the Supplemental Materials (Table S4).

### Statistical analysis

Data are expressed as means ± SEM. Statistical analysis was performed with GraphPad Prism 6.0 software. Comparisons between two groups were analyzed by Student’s *t-test*. More than two groups were compared using a one-way factorial analysis of variance (ANOVA), followed by Student’s *t-test*. The nature of errors bars in graphical representations and the number of biological replicates (n) is indicated in the corresponding figure legend.

### Data availability

All data necessary for confirming the conclusions presented are within the article. File S1 contains supplemental data to support the conclusions in this article. Supplemental material available at Figshare: https://doi.org/10.25387/g3.7502405.

## Results

### Generation of transgenic H11-albumin-rtTA mice

The *H11* locus is located in an intergenic region, flanked by the two genes *Eif4enif1* and *Drg1* (Figure 1A). The *H11-albumin-rtTA* donor plasmid, in which the *albumin-rtTA* cassette was flanked by two 1.3 kb *H11* homologous arms, was used as the template for CRISPR/Cas9-mediated homologous recombination (HR). The *albumin-rtTA* cassette contained *rtTA* coding sequence and *poly A* elements directed by the *albumin* promoter. sgRNA was designed to target the *H11* locus. Then, the *Cas9* mRNAs, sgRNA, and *H11-albumin-rtTA* donor plasmid were co-microinjected into zygotes, which were subsequently transferred to pseudopregnant females (Figure 1A).

After microinjection and embryo transfer, we obtained 22 pups. Two pairs of primers (upstream and downstream) were designed to detect HR-mediated *albumin-rtTA* cassette insertion. The primers used for PCR amplification are indicated (Figure 1A) and are detailed in supplementary Table 2. The primers were designed outside of the homologous arm to exclude random insertion. Thus, only genomic DNA with correct insertion of the *albumin-rtTA* cassette produced the desired PCR products (Figure 1B). PCR and sequencing showed that 8 pups (36%) harbored the correct *albumin-rtTA* insertion. We named the HR-mediated *albumin-rtTA* insertion mice as *H11-albumin-rtTA* mice.

### Liver-specific inducible transgene expression in Dox-treated H11-albumin-rtTA mice

**Figure 2:**
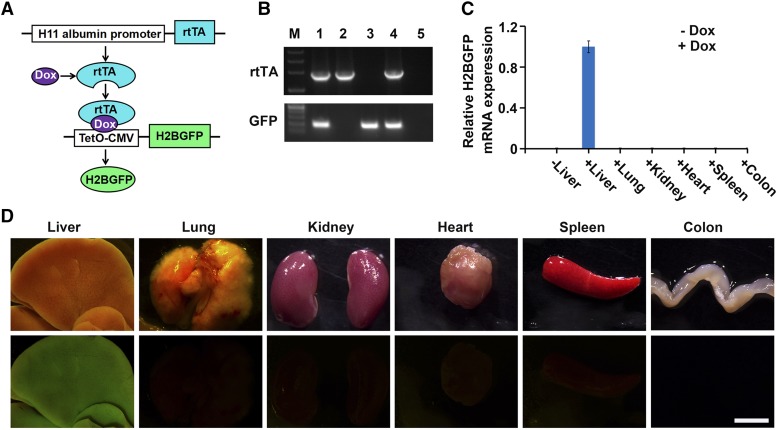
Dox-inducible *H2BGFP* reporter gene expression in different organs of *H11-albumin-rtTA/TetO-H2BGFP* double-transgenic mice. (A) Schematic diagram of induced *H2BGFP* expression in the *H11-albumin-rtTA/TetO-H2BGFP* mice after Dox treatment. The rtTA protein binds with Dox and forms a complex that initiates transcription of a H2BGFP fusion protein construct positioned downstream of a tetracycline inducible promoter (TetO-CMV). (B) PCR genotyping of mice. The positive transgenic mice had a 1.9 kb product for *rtTA*; *H2BGFP* generated a 191 bp product. Lanes 1 and 4 were transgene^+^ mice; lanes 2, 3, and 5 were transgene^-^ mice. (C) qRT-PCR analysis of the relative expression of *H2BGFP* mRNA in different organs dissected from double transgenic mice treated with 1mg/ml Dox (+Dox) or water (-Dox) for 48 h (*H2BGFP* expression was normalized to *Gapdh* (n = 4)). Error bars show mean ± SEM (n = 4). (D) Light images (top panels) and whole mount fluorescence microscopy (bottom panels) analyses of H2BGFP protein in the Dox treated *H11-albumin-rtTA/TetO-H2BGFP* mice. Scale bar: 5 mm.

To examine the suitability of using this newly introduced construct for the inducible and tissue-specific expression of transgenes, the *H11-albumin-rtTA* mice were crossed with *TetO-H2BGFP* mice to generate *H11-albumin-rtTA/TetO-H2BGFP* double-transgenic mice (Figure 2A). The genotypes of all mice were confirmed by PCR analysis of tail DNA (Figure 2B). Adult *H11-albumin-rtTA/TetO-H2BGFP* mice were given drinking water supplemented with 1mg/ml Dox for two days, and qRT-PCR and whole mount fluorescence microscopy were used to characterize the induction of H2BGFP reporter expression in a variety of mouse tissue types (Figure 2C-D). In the absence of Dox treatment, no *H2BGFP* mRNA expression was detected in the liver of *H11-albumin-rtTA/TetO-H2BGFP* mice. The expression of both *H2BGFP* mRNA transcripts and H2BGFP protein was restricted to the liver of Dox-treated *H11-albumin-rtTA/TetO-H2BGFP* mice (*i.e.*, no expression was detected in heart, spleen, colon, lungs, or kidneys).

In addition, we examined expression of hepatocyte differentiation marker albumin and of GFP in the liver of Dox-treated *H11-albumin-rtTA/TetO-H2BGFP* mice. Our immunofluorescence results demonstrated that the GFP and albumin proteins were co-expressed within the same liver cells (Figure S1). Moreover, the expression of GFP was restricted to well-differentiated hepatocytes. Notably, HE staining revealed no obvious histological or morphological differences between untreated and Dox-treated *H11-albumin-rtTA/TetO-H2BGFP* mice (Figure S2). These results collectively establish that our new site-specific knock-in *H11-albumin-rtTA* transgenic mouse model can be used as a valuable tool for the Dox-inducible and liver-specific expression of a given transgene-of-interest.

### Reporter gene induction and extinction in Dox-treated H11-albumin-rtTA mice

**Figure 3:**
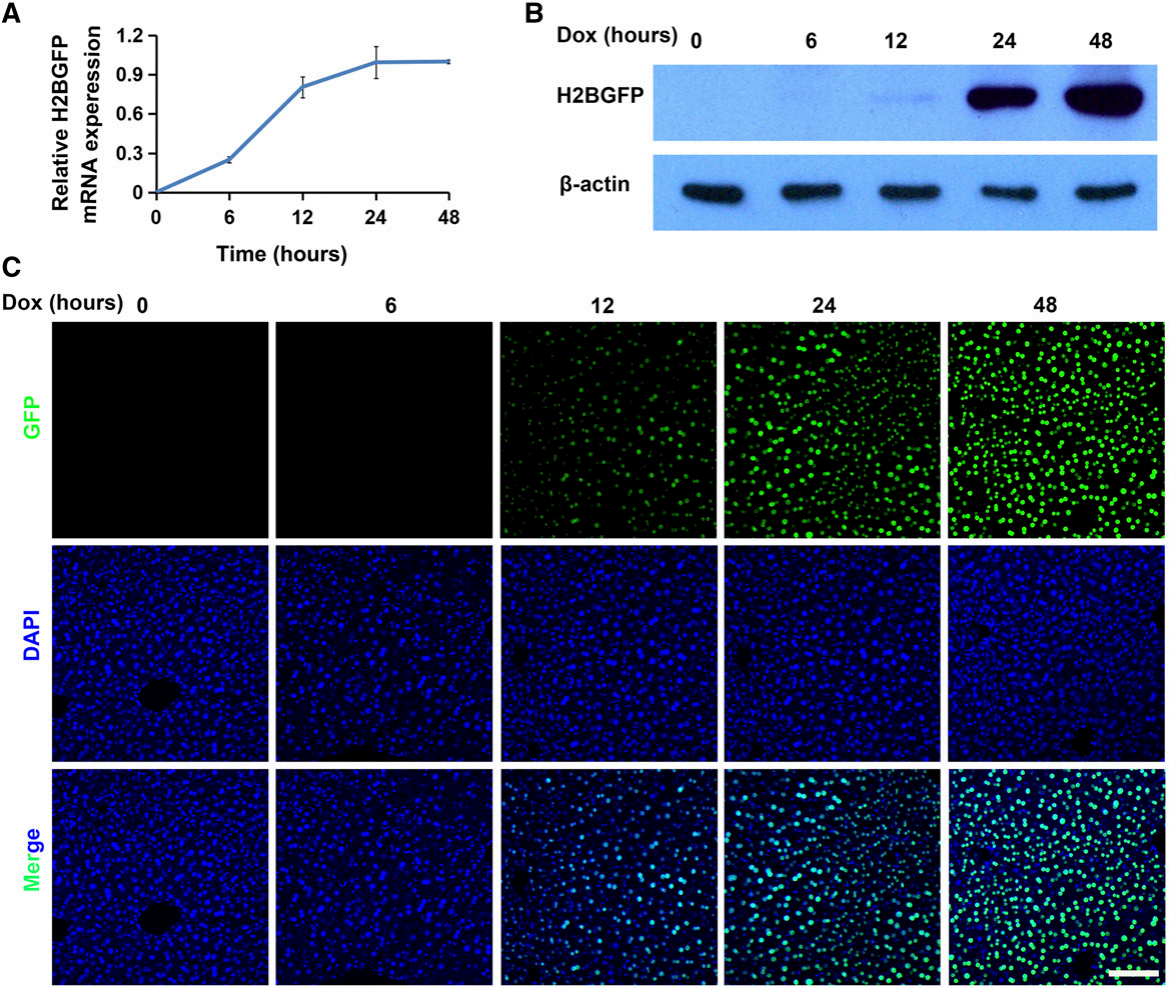
H2BGFP expression in Dox-treated *H11-albumin-rtTA/TetO-H2BGFP* mice. (A) qRT-PCR analysis of the relative expression of *H2BGFP* mRNA in response to Dox exposure (*H2BGFP* expression was normalized to *Gapdh* (n = 4)). Error bars show mean ± SEM (n = 4). (B-C) Western blotting and immunofluorescence analyses of H2BGFP protein obtained from livers of mice treated with Dox over a 48 h period. GFP staining (green); DAPI nuclear counterstaining of DNA (blue). Scale bar: 100 μm.

We measured reporter gene expression in Dox-treated *H11-albumin-rtTA/TetO-H2BGFP* mice over a 48 h period. qRT-PCR analysis of *H2BGFP* mRNA accumulation following Dox exposure revealed that transgene expression had started by 6 h, continued rising to 24 h, and then remained steady up to end of the experiment at 48 h (Figure 3A). Western blotting and immunofluorescence results demonstrated that obvious H2BGFP protein expression could be detected at 12 h and reached its maximum detected level at 48 h (Figure 3B-C).

**Figure 4:**
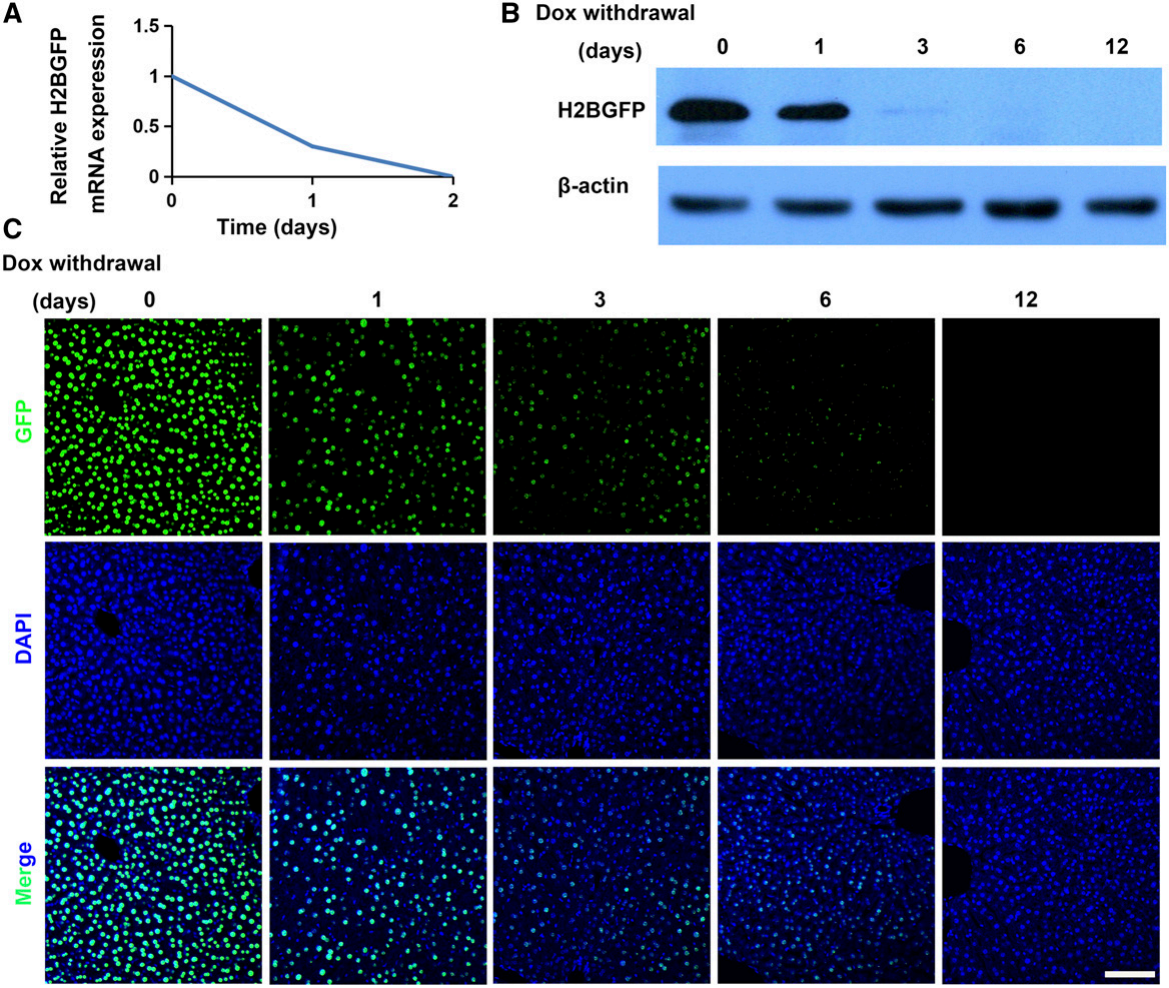
Extinction of H2BGFP expression after stopping Dox-treatment of *H11-albumin-rtTA/TetO-H2BGFP* mice. (A) qRT-PCR analysis of the relative expression of *H2BGFP* mRNA in response to Dox cessation (*H2BGFP* expression was normalized to *Gapdh* (n = 4)). (B-C) Western blotting and immunofluorescence analyses of protein obtained from livers of treated mice (from start of Dox withdrawal to day 12). GFP staining (green); DAPI nuclear counterstaining of DNA (blue). Scale bar: 100 μm.

We also examined how cessation of Dox treatment affected transgene expression in *H11-albumin-rtTA/TetO-H2BGFP* mice that had already been treated with Dox for 48 h. qRT-PCR analysis showed that *H2BGFP* mRNA levels diminished rapidly within 1 day of stopping Dox treatment and no *H2BGFP* transcripts could be detected by the second day (Figure 4A). Western blotting and immunofluorescence analyses showed stopping Dox treatment resulted in a significant decrease in H2BGFP protein levels by day 3, with H2BGFP being nearly undetectable by day 6; note that GFP is highly stable, so the low levels observed on day 6 may represent residual protein that was translated during the Dox-induction phase (Figure 4B-C). Collectively, these results highlight the tight temporal regulation of Dox-inducible transgene expression that can be achieved using the *albumin-rtTA* element that we introduced in the *H11* locus in our new site-specific knock-in transgenic mouse model.

### Combining the Cre/loxP recombination system with H11-albumin-rtTA mice

**Figure 5:**
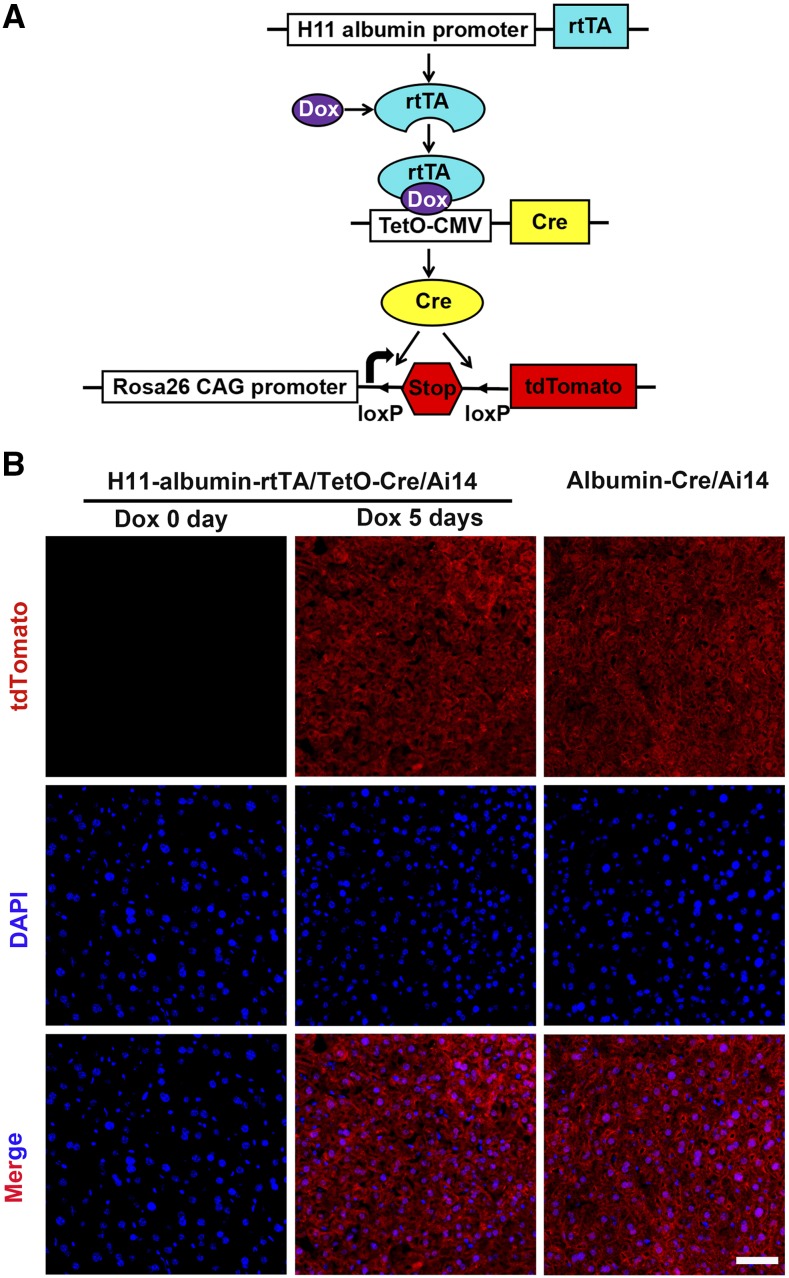
Dox-inducible tdTomato expression in the livers of *H11-albumin-rtTA/TetO-Cre/Ai14* mice. (A) Schematic diagram of induced tdTomato expression in the *H11-albumin-rtTA/TetO-Cre/Ai14* triple transgenic mice after Dox treatment. The rtTA protein binds Dox and forms a complex that initiates transcription of Cre recombinase downstream of a TetO-CMV. Subsequently, Cre excises the loxP-flanked Stop sequence from the *Rosa26* locus, inducing tdTomato expression. (B) Immunofluorescence analysis of tdTomato protein expression in the adult *H11-albumin-rtTA/TetO-Cre/Ai14* mice which were treated with or without 1 mg/ml Dox for 5 days (Dox 0 day, left panel; Dox 5 days, middle panel) and in the *Albumin-Cre/Ai14* mice (right panel). tdTomato staining (red); DAPI nuclear counterstaining of DNA (blue). Scale bar: 50 μm.

The Cre/loxP recombination system, which enables conditional DNA recombination, is a well-established research tool that is especially popular for experiments with transgenic mice (*e.g.*, *albumin-Cre* mice ubiquitously in studies of liver development and disease (Cui *et al.* 2016)). Thus, we tested if we could implement Cre/loxP conditional DNA recombination technology with our mice by crossing Dox-inducible *H11-albumin-rtTA* mice with *TetO-Cre* and *Ai14* mice to generate *H11-albumin-rtTA/TetO-Cre/Ai14* triple transgenic mice. Specifically (Figure 5A), *Ai14*, a knock-in allele of the *Rosa26* locus that allows high-level ubiquitous expression of the red fluorescent protein tdTomato downstream of a loxP-flanked transcriptional stop signal: in the presence of Cre, the stop codon is excised, and tdTomato expression proceeds (Madisen *et al.* 2010).

Adult *H11-albumin-rtTA/TetO-Cre/Ai14* triple transgenic mice were given drinking water supplemented with 1 mg/ml Dox for 0, 3, or 5 days (Figure S2). Whole mount fluorescence microscopy and immunofluorescence analysis were used to characterize the induction of dTomato reporter expression (Figure 5B and Figure S2), and we found that, in the absence of Dox treatment, no dTomato signal was detected in the livers of *H11-albumin-rtTA/TetO-Cre/Ai14* mice. The mount fluorescence analysis showed that the dTomato signal levels in the livers *H11-albumin-rtTA/TetO-Cre/Ai14* mice became stronger as the length of Dox treatment increased (Figure S3). Notably, highlighting the very high Cre-mediated recombination efficiency achieved with this Dox-inducible system for conditional transgene expression in our *H11-albumin-rtTA/TetO-Cre/Ai14* mice, we found that the tdTomato signal of the *H11-albumin-rtTA /TetO-Cre/Ai14* mice treated with Dox for 5 days was apparently as strong as the signal in the *albumin-Cre/A14* mice, which express tdTomato in the liver constitutively (Figure 5B and Figure S3).

### Inducible and tissue-specific transgene expression in embryonic and neonatal mice

**Figure 6:**
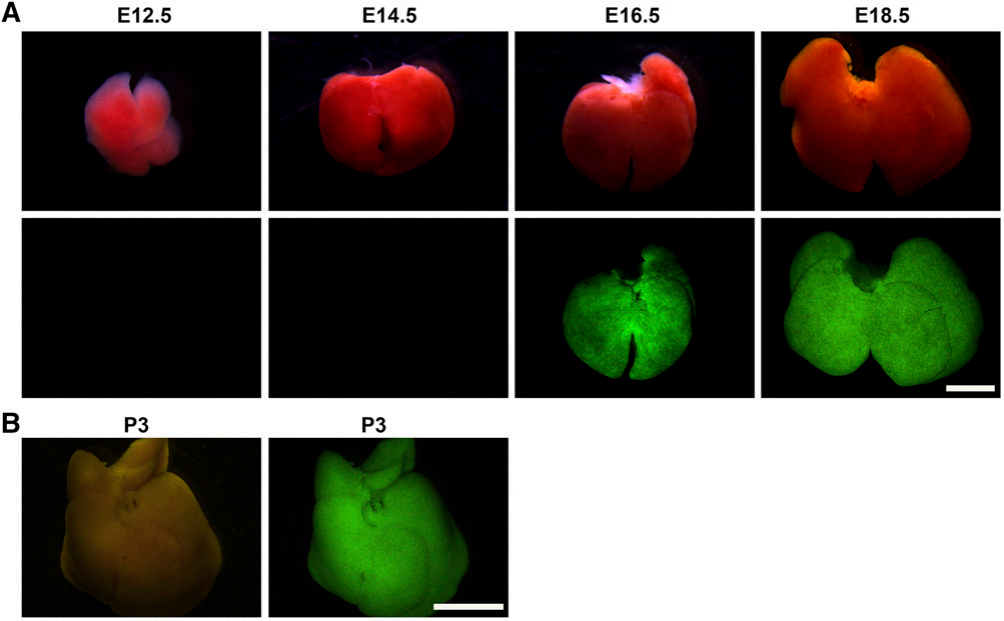
Dox-inducible H2BGFP reporter gene expression in the livers of embryonic and neonatal *H11-albumin-rtTA/TetO-H2BGFP* double-transgenic mice. (A) Light images (top panels) and whole mount fluorescence microscopy (bottom panels) analysis of H2BGFP protein expression in the livers of embryonic mice at successive developmental stages. H2BGFP expression in the liver at E12.5, E14.5, E16.5, and E18.5. Scale bar: 2 mm. (B) Light image (left panel) and whole mount fluorescence microscopy (right panel) analysis of H2BGFP protein expression in the livers of P3 mice. Scale bar: 5 mm.

Having determined that our new site-specific knock-in *H11-albumin-rtTA* transgenic mouse model can be used with adult mice, we next explored its use in developing mouse embryos. Dox treatment of (1mg/ml) pregnant female mice was initiated upon detection of the vaginal plug, and embryos were analyzed by whole mount fluorescence microscopy analysis at E12.5, E14.5, E16.5, and E18.5 in *H11-albumin-rtTA/TetO-H2BGFP* mice. No GFP signal was detected before E16.5; strong GFP signal was observed at E18.5 (Figure 6A). We also conducted an experiment in which neonatal *H11-albumin-rtTA/TetO-H2BGFP* mice were administered an oral gavage of 20 μl of Dox (1 mg/ml) at P1 for two consecutive days (12 hr a time), which revealed strong liver-specific GFP expression at P3 (Figure 6B). Together, these results demonstrate that our new site-specific knock-in *H11-albumin-rtTA* transgenic mouse model can be used for Dox-inducible and tissue-specific expression of transgenes-of interest in both embryos and neonatal pups.

## Discussion

In this study, we developed a new *H11* site specific knock-in transgenic mouse model in which *albumin-rtTA* elements were inserted into the endogenous *H11* locus via homologous recombination. The use of this locus to drive generalized gene expression addresses many of the problems associated with random DNA insertion integration, such as unstable insertion, insertion-site side effects, and low insertion efficiency (Sadelain *et al.* 2011). To date, the *Rosa26* locus has been popularly used as a transgene insertion site; it causes no apparent adverse effects and permits stable gene expression (Sukup-Jackson *et al.* 2014). Our study confirms the previously reported findings about *H11* as an attractive locus for transgene insertion (Hippenmeyer *et al.* 2010; Zhu *et al.* 2014; Ruan *et al.* 2015). Previous studies in mice have established that genes inserted into the *H11* locus can display robust, ubiquitous expression that is apparently higher than the expression levels achieved with other commonly used ubiquitously loci, including *Rosa26* (Hippenmeyer *et al.* 2010; Tasic *et al.* 2011; Tasic *et al.* 2012).

Our *H11-albumin-rtTA* mice will be particularly useful for studies of the liver, as we confirmed that *H2BGFP* expression is specifically localized to the liver of Dox-treated *H11-albumin-rtTA/TetO-H2BGFP* double-transgenic mice and verified that *albumin* promoter can activate rtTA expression in a Dox-dependent manner. Previous studies of albumin expression in embryonic mice reported that *albumin* mRNA was initially detected at E10.5 and then increased continuously and reached maximal expression in adult livers (Jochheim *et al.* 2004). However, and indicating an obvious discrepancy, we found in the *H11-albumin-rtTA/TetO-H2BGFP* mice the GFP expression level driven by the albumin promoter was undetectable at E12.5 and E14.5; we found that newly conceived *H11-albumin-rtTA* embryos that were treated with Dox were expressing GFP reporter protein by E16.5. Further, Hayhurst *et al.* (2001) reported that *albumin-Cre* transgenic mice express Cre exclusively in the postpartum liver (Yakar *et al.* 1999; Hayhurst *et al.* 2001). Thus, it appears that the strength of *albumin* promoter activity in embryonic Dox-inducible *H11-albumin-rtTA* mice is significantly higher than in *albumin-Cre* transgenic mice. These discrepancies may be related to the higher efficiency of inducing gene expression in transgenic mice generated by site-specific insertion. Nevertheless, it is clear that our *H11-albumin-rtTA* mice can be widely used to manipulate target gene expression in the livers of later embryonic and neonatal mice.

In some mechanistic studies, researchers need to manipulate gene expression in more than one cell type or in the same cell type but at different ages. This can be achieved for example by combining Tet-on inducible mouse models and the widely deployed Cre/loxP system, which has become a standard method of choice for cell-type or tissue-specific gene knockout in mice (Xue *et al.* 2014). In recent years, there have been many studies that used targeted Cre-mediated conditional gene expression in the liver (He *et al.* 2017; Chao *et al.* 2018). Our new Dox-inducible *H11-albumin-rtTA* mice add a flexible new option for researchers to use in their experimental designs: we successfully confirmed that Dox-induced *albumin*-driven Cre expression in the is very high in transgenic mice. When researchers need to study a liver-specific gene in mice that already harbor engineered sequences at their *Rosa26* locus, *H11-albumin-rtTA* mice will be an excellent experimental option, especially considering the hepatocyte-specific expression driven by the albumin promoter. For example, for liver studies that need to use some particular line of knock-in mice (*e.g.*, *Rosa26-LSL* (*loxP-Stop-loxP*)*- given-gene-of-interest*) and that also want to use *TetO-Cre* mice, we could use our new *H11-albumin-rtTA* mice to generate *H11-albumin-rtTA/TetO-Cre/Rosa26-LSL-given-gene-of-interest* mice. Transgenic animals are now recognized as invaluable research tools for studying human disease, and beyond highlighting the utility of the *H11* locus generally, our study establishes that these new *H11-albumin-rtTA* mice enable unprecedently temporal and spatial control of the expression for genes-of-interest in the liver.
